# Optimizing the Ultrasound Tongue Image Representation for Residual Network-Based Articulatory-to-Acoustic Mapping

**DOI:** 10.3390/s22228601

**Published:** 2022-11-08

**Authors:** Tamás Gábor Csapó, Gábor Gosztolya, László Tóth, Amin Honarmandi Shandiz, Alexandra Markó

**Affiliations:** 1 Department of Telecommunications and Media Informatics, Budapest University of Technology and Economics, H-1117 Budapest, Hungary; 2 ELRN-SZTE Research Group on Artificial Intelligence, H-6720 Szeged, Hungary; 3 Institute of Informatics, University of Szeged, H-6720 Szeged, Hungary; 4 MTA-ELTE Lendület Lingual Articulation Research Group, H-1088 Budapest, Hungary

**Keywords:** speech processing, ultrasound imaging, deep learning

## Abstract

Within speech processing, articulatory-to-acoustic mapping (AAM) methods can apply ultrasound tongue imaging (UTI) as an input. (Micro)convex transducers are mostly used, which provide a wedge-shape visual image. However, this process is optimized for the visual inspection of the human eye, and the signal is often post-processed by the equipment. With newer ultrasound equipment, now it is possible to gain access to the raw scanline data (i.e., ultrasound echo return) without any internal post-processing. In this study, we compared the raw scanline representation with the wedge-shaped processed UTI as the input for the residual network applied for AAM, and we also investigated the optimal size of the input image. We found no significant differences between the performance attained using the raw data and the wedge-shaped image extrapolated from it. We found the optimal pixel size to be 64 × 43 in the case of the raw scanline input, and 64 × 64 when transformed to a wedge. Therefore, it is not necessary to use the full original 64 × 842 pixels raw scanline, but a smaller image is enough. This allows for the building of smaller networks, and will be beneficial for the development of session and speaker-independent methods for practical applications. AAM systems have the target application of a “silent speech interface”, which could be helpful for the communication of the speaking-impaired, in military applications, or in extremely noisy conditions.

## 1. Introduction

Speech is used in our everyday human–computer interfaces when interacting with mobile or fixed electronic devices. Future speech interfaces will go beyond current human–machine communication systems because speech has several drawbacks: (1) it can be easily captured by a third party; (2) speech communication is problematic for the speaking-impaired (e.g., patients after laryngectomy); (3) speech understanding degrades rapidly in noisy environments.

There has been an increased interest in the analysis, processing, prediction, and synthesis of biosignals in the speech processing community. Such biosignals include: the speech waveform, information about the articulators (larynx, tongue, lips, teeth, etc.), neural pathways, or the brain itself. These biosignals can be used in scenarios such as articulatory-to-acoustic mapping (AAM) or acoustic-to-articulatory inversion (AAI). Such biosignals can typically be recorded with some external sensor or specific device, and processing this data causes various challenges. In the AAM field, articulatory data (i.e., information about the movement of the articulatory organs) are recorded while the subject is speaking, and machine learning methods (nowadays, typically deep neural networks (DNNs)) are applied for predicting the speech signal, while the network is conditioned on the articulatory input. Systems that can perform the automatic articulatory-to-acoustic mapping are often referred to as “silent speech interfaces” (SSIs) [1,2,3], with the final aim of a target application where silent (mouthed) articulation can be converted to audible speech. Such an SSI could be helpful for the communication of the speaking-impaired, in military applications, or in extremely noisy conditions.

In the area of AAM, several different types of articulatory acquisition equipments have been used, including ultrasound tongue imaging (UTI) [4,5,6,7,8,9,10,11,12,13,14,15,16,17,18,19,20,21,22], electromagnetic articulography (EMA) [23,24,25,26,27], permanent magnetic articulography (PMA) [28,29], surface electromyography (sEMG) [30,31,32], electro-optical stomatography (EOS) [33], lip video [5,6,34,35,36], continuous-wave radar [37], or multimodal combination [38]. There are basically two distinct methods of SSI solutions, namely “direct synthesis” and “recognition-and-synthesis” [2]. In the first case, the speech signal is generated without an intermediate step, directly from the articulatory data, typically using vocoders [4,7,9,11,12,15,16,17,25,26,29,30,31]. In the second case, silent speech recognition (SSR) is applied on the biosignal, which extracts the content spoken by the person (i.e., the result is text). This step is then followed by text-to-speech (TTS) synthesis [5,6,10,23,24,28,32,33]. The drawback of the SSR+TTS approach might be that the errors made by the SSR component inevitably appear as errors in the final TTS output [2], and also that it causes a significant end-to-end delay. Furthermore, any information related to speech prosody is totally lost, while several studies have showed that certain prosodic components may be estimated reasonably well from the articulatory recordings (e.g., energy [11] and pitch [12]). Depending on the use-case scenario, the two approaches may have various advantages; for example, the smaller delay with the direct synthesis approach might enable conversational use and potential research on human-in-the-loop scenarios.

In this study, we focus on ultrasound tongue images as the articulatory input, with the direct synthesis approach used for AAM.

### 1.1. Representations of Ultrasound Tongue Images

**Figure 1:**
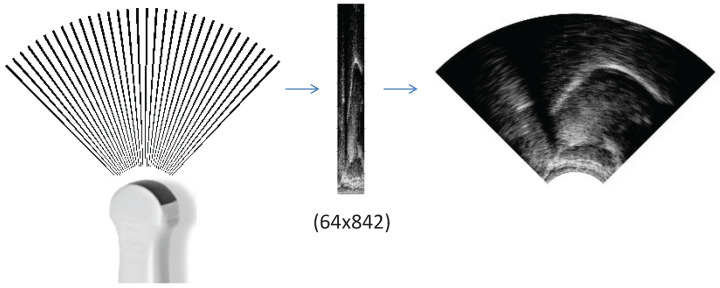
Ultrasond tongue image representations: raw scanlines during recording (**left**), array of raw scanline data (**middle**), and a wedge-formatted image (**right**).

For investigating the tongue movement using ultrasound, a B-mode scan is typically used with a (micro)convex transducer [39]. In a real-time B-scan ultrasound transducer, a row of identical piezoelectric crystals emit sound waves and receive their reflected echoes (for an illustration, see the left-hand side of Figure 1). The received echoes are converted to an electrical signal, and are then sent to the internal computer of the ultrasound machine. The internal computer reconstructs the returning echoes into a 2D grayscale image usually shaped like a 90–120 degree wedge (see the right-hand side of Figure 1). Typically, during recordings, a midsagittal orientation is maintained with the shadows of the jaw and the hyoid bones visible at opposite sides of the scan wedge [39]. For linguistic studies, manual tracing or the automatic tracking of the tongue is frequently performed [40,41], but, for articulatory-to-acoustic mapping purposes, such a contour extraction is not typically used.

In the first AAM studies that had ultrasound images for recording the articulatory movement, it was not possible to gain access to the raw echo data due to the restrictions of the equipment. Instead, the ultrasound scanlines were interpolated and organized as a “fan-shaped”/“wedge” representation, as described above. In the earliest UTI-based direct synthesis study by Denby et al. [4], the ultrasound images (recorded at 30 fps) were first reduced to a 14 by 40 grid and automatic contour tracking was carried out on the fan-shaped data to reduce dimensionality. A few years later, Hueber et al. [6] used fan-shaped images (with an Aloka SSD-1000 machine), but post-processing algorithms, such as image averaging and speckle reduction, were disabled. After this, with an analog system, an NTSC video was created, limiting the time resolution to 29.97 Hz fps. In their next experimental setup [5,7,9], a Terason T3000 ultrasound system was used with a dedicated software to record the wedge-shaped articulatory data at 320×240 pixels and 60 fps, doubling the time resolution. The fan-shaped ultrasound images were resized to 64×64 pixels and the EigenTongues decomposition technique [42] was applied for dimension reduction, keeping the first 30 coefficients. In the latest relevant study from the same research group [10], the ultrasound images were resized to 32×32 pixels, and these images were used with CNNs (without EigenTongues compression). Similarly, a 320×240 pixels ultrasound video was recorded for the Silent Speech Challenge (SSC) dataset [14]. Wei et al. [8], with an unspecified system, used a fan-shaped 64×48 pixels UTI input (compressed with PCA and autoencoders) for AAM and AAI. Kimura et al. [18] used a CONTEC CMS600P2 system and a display-digitizing unit for converting the signal sent to the display to a 30 fps MPEG-4 movie file, and resized the fan-shaped images to 128×128 pixels for the AAM input. In their next study [43], interpolated ultrasound videos were recorded with a resolution of 640×445 pixels. In most of the above studies, classical image processing of the ultrasound input is not performed, and the feature extraction is left to the DNN. This is similar to how other modalities are processed in related tasks such as lip images [34], MRI [44], or EMA [27].

In our earlier studies on ultrasound-based articulatory-to-acoustic mapping, we used raw scanline data as the input of the DNNs, recorded using a “Micro” system (developed by Telemed Ltd., Vilnius, Lithuania, and distributed by Articulate Instruments Ltd., Musselburgh, UK), a 2–4 MHz/64 element 20 mm radius convex ultrasound transducer at 80–85 fps [11,12,13,15,16,17,20,21]. In [11,12,13], data from a single female speaker were used, and the raw echo-returns of 64×946 were resized to 64×119 pixels using a bicubic interpolation. In [17], four speakers were used, and the raw images of 64×842 pixels were resized to 64×128. Instead of using the full raw scanline data, in [11], we investigated correlation-based feature selection, and, in [16], we tested the applicability of autoencoders for dimensionality reduction. Besides the above works by our research group, there were only a few studies that used raw scanlines. Ribeiro et al. [45] applied a raw ultrasound for the classification of phonetic segments. Here, 63×412 echo-return data (recorded using Ultrasonix SonixRP) were utilized as the input of DNNs and CNNs, and the raw data input was compared with PCA and 2D-DCT-based compression. A subsequent study [46] applied the raw scanlines of the “Micro” system, resized to 63×128 pixels.

The advantage of fan-shaped data is that they correspond to the physical/spatial orientation of the speaking organs of the subject; therefore, comparisons across sessions and speakers are relatively easy. Another benefit can be that CNNs might process the wedge-shaped data easier as they do not contain nonlinear distortions. On the other hand, the advantage of raw scanline data is that they can be acquired directly from the ultrasound equipment, without any postprocessing. Therefore, feature extraction can be left up to the machine learning algorithms. However, the disadvantage is that, because of the convex transducer, the raw data do not correspond to the original mid-sagittal slice, and non-linear interpolation is necessary to transform into real-world orientation. Therefore, a comparison across sessions and speakers using the raw scanline data is a challenge.

### 1.2. Contributions of This Paper

In our previous studies, we hypothesized that the use of a raw scanline ultrasound always results in lower errors during the prediction of spectral or excitation parameters [11,12,13,15,16,17]. However, this hypothesis was never tested explicitly (neither by us, nor by other research groups). In the current paper, we compared raw scanline data with the wedge-formatted ultrasound tongue image input for articulatory-to-acoustic mapping, applying deep neural networks. Furthermore, we investigated the effect of reducing the input image size.

## 2. Materials and Methods

### 2.1. Data Acquisition

**Figure 2:**
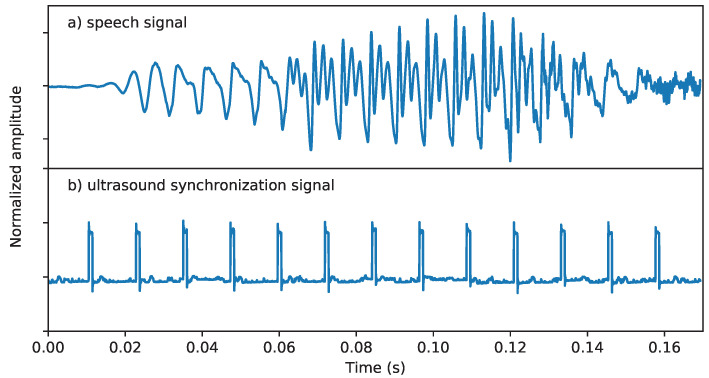
Ultrasound synchronization signal: the rising edge of the pulses indicates the capture time of ultrasound images.

The same dataset was used as in our previous studies [17,20]. Several Hungarian male and female subjects with normal speaking abilities were recorded while reading sentences aloud (altogether, 209 sentences each), of which, a female speaker (#048) was chosen for the current study. The tongue movement was recorded in midsagittal orientation using the “Micro” ultrasound system (Articulate Instruments Ltd.) with a 2–4 MHz/64 element 20 mm radius convex ultrasound transducer at 81.67 fps. The speech signal was recorded with a Beyerdynamic TG H56c tan omnidirectional condenser microphone. At the time of capturing an ultrasound frame, the “Micro” equipment generates a pulse at the “frame sync” output, which was digitized together with the speech signal with an M-Audio—MTRACK PLUS external sound card at 22 050 Hz (see Figure 2). The ultrasound data and the audio signals were synchronized using a custom tool that is looking at the rising edge of the peaks in the "frame sync" signal. More details about the recording set-up can be found in [11,17]. The overall duration of the recordings was approximately 15 min, which was partitioned into training, validation, and test sets in an 85:10:5 ratio.

### 2.2. Input 1: Ultrasound as Raw Scanlines (UTIraw)

**Figure 3:**
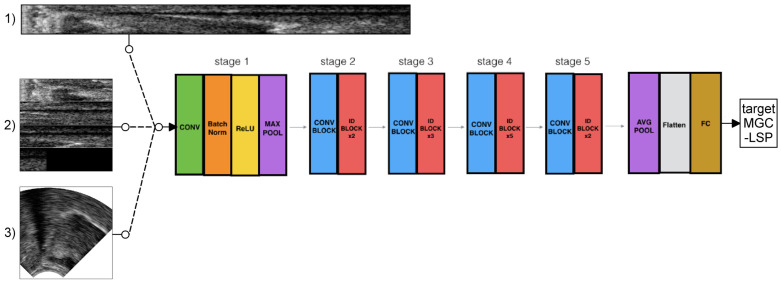
ResNet-50 architecture for articulatory-to-acoustic mapping using ultrasound tongue image (raw scanline vs. wedge) input and MGC-LSP target. ResNet image adopted from https://towardsdatascience.com/understanding-and-coding-a-resnet-in-keras-446d7ff84d33, accessed date: 11 May 2020.

In the first case, the raw scanline data (64×842 pixels, Figure 3/1) of the ultrasound were used. To check the optimal image resolution, they were further resized to 64 × 421, 64 × 210, 64 × 105, 64 × 53, 64 × 26, and 64 × 13 pixels using bicubic interpolation (with the skimage.transform function). The resized raw images served as the input of the deep neural networks, which can be seen in Figure 3 and will be introduced in Section 2.6.

### 2.3. Input 2: Ultrasound as Raw Scanlines, Reshaped to Square (UTIraw-Padding)

In the second case, the scanline data (64×842 pixels) of the ultrasound were used, after being transposed to a 512 × 512 square for ResNet input (see Figure 3/2). To check the optimal image resolution, they were further resized to 256 × 256, 128 × 128, 64 × 64, 32 × 32, 16 × 16, and 8 × 8 pixels using bicubic interpolation (with the skimage.transform function).

### 2.4. Input 3: Ultrasound as a Wedge-Shape (UTIwedge)

In the third case, the raw scanline data (left-hand side of Figure 1) were interpolated to achieve a wedge-shape. For this, we used the pcolormesh function of matplotlib to smooth and interpolate the data for a continuous wedge-shape, including aliasing (right-hand side of Figure 1). The necessary details for the interpolation (e.g., angle between scanlines, zero offset) were extracted from the AAA software (V219.08, Articulate Instruments Ltd.) that was used for the recordings. The generated image sequences (840 × 510 pixels) were saved to MP4 video using ffmpeg, keeping the original scaling of the pixel values. After this, the middle of the images was cropped to a 512 × 512 square box (region of interest), and this was used as the input of the ResNet (see Figure 3/3). The further image resizing steps were the same as those for the raw scanline data in Section 2.3, i.e., resized to 256 × 256, 128 × 128, 64 × 64, 32 × 32, 16 × 16, and 8 × 8 pixels using bicubic interpolation (with the skimage.transform function).

### 2.5. Target: Spectral Features of the Vocoder

To create the speech synthesis targets, the speech recordings were analyzed using mel-generalized log spectral approximation (MGLSA) [47] at a frame shift of 22,050 Hz/81.67 fps = 270 samples in order to be synchronous with the ultrasound data. As shown in Figure 2, this was achieved using the hardware sync output of the “Micro” equipment. This resulted in 25-dimensional spectral features (mel-generalized cepstrum–line spectral pair representation (MGC-LSP)) [48]. The vocoder spectral parameters served as the training targets of the DNNs, similarly to our earlier experiments in articulatory-to-acoustic mapping [11,17].

### 2.6. Training of Deep Neural Networks

We applied the ResNet-50 network [49] for the deep learning experiments. In our earlier studies, we either used fully connected deep neural networks [11,12], convolutional networks [15,17,20], LSTMs [15], 3D-CNNs [21], or GANs [22], but here, we opted for a more advanced network. The advantage of ResNet is that, by using skip connections, deeper convolutional networks can be trained than with simple DNNs or CNNs. By using ResNet-50, the network is spatially deep enough to capture most information from the ultrasound-based articulatory data. As ResNet was originally developed for image classification, the original output layer is “softmax”, which was replaced here by a “linear” activation for the current regression task.

For all cases, we trained a speaker-specific ResNet model using the training data (180 sentences). Altogether, 21 networks were trained (3 data representations × 7 image sizes × 1 speaker). The cost function applied for the MGC-LSP regression task was the normalized mean-squared error (NMSE), and the optimizer was ADAM. We trained the network using backpropagation, and applied early stopping to avoid over-fitting. The network was trained at most for 100 epochs, but the training was stopped when the validation loss did not decrease within 10 epochs.

## 3. Results

After training the above ResNet models, we evaluated them by comparing the input image representations and the output spectral features.

### 3.1. Demonstration Samples

**Figure 4:**
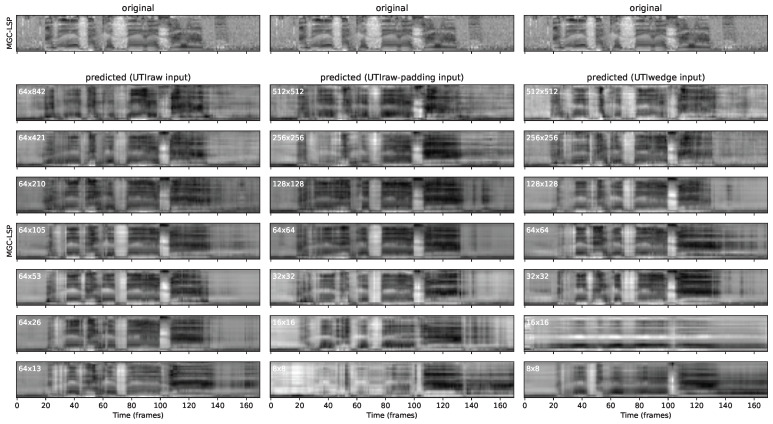
Demonstration samples: predicted MGC-LSP spectral features as a function of input image representation and size. Sentence: “Az Északi szél és a Nap”.

A sample Hungarian sentence (not being present in the training data) was chosen for demonstrating how the systems deal with the prediction of MGC-LSP spectral parameters. Figure 4 shows the output spectral features with the three input representations and seven image sizes.

In the first column, we can compare the results when using ultrasound as a raw scanlines input between 64×842–64×13 pixels. The predicted spectrograms follow the original sentence for the most part, but we can observe some artifacts: in the case of large input sizes (64×842, 64×421 and 64×210), the spectrogram is oversmoothed (i.e., formants are only weakly visible); and with a very small input size (64×13), unwanted frequency components appear at the end of the sentence, after frame 130. The remaining three figures in the middle (64×105, 64×53, and 64×26) seem to be the closest to the original spectrogram.

The second column shows the results when using the ultrasound of raw scanlines input, reshaped to a square, between 512×512–8×8 pixels. The tendencies are similar to the first column: the largest (512×512) and smallest (8×8) images cause oversmoothing, whereas those in between follow the spectral features or the original sentence with finer details. Interestingly, the 128×128 image size resulted in some distortion at the end of the sentence, between frames 140–160.

In the third column of Figure 4, we can see the effect ofthe ultrasound as a wedge-shape when used as an input of the ResNet, again between 512×512–8×8 pixels. The middle images sizes (128×128, 64×64, and 32×32) resulted in a relatively well-predicted spectrogram between frames 20–140; but after frame 140, distortion is visible in the case of 64×64. In the case of this demonstration sentence, the spectral prediction with 16×16 is extremely weak and almost constant, whereas in the case of the 8×8 image size, the formant movements of the original spectrogram are at least roughly visible.

Overall, the best MGC-LSP spectrogram predictions could be achieved with input image sizes of 64×53, 64×64, and 32×32 pixels on this single demonstration sentence. To obtain more general evaluations, we measured errors on the whole validation set, which will be introduced in the next section.

### 3.2. Comparison of Raw Scanline Data and Wedge Format

**Figure 5:**
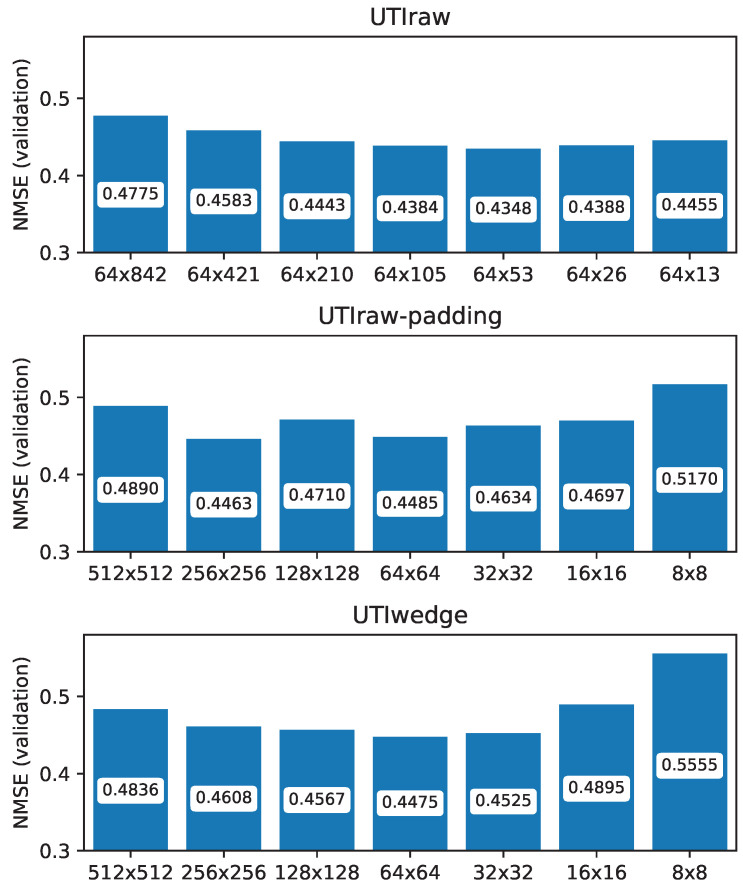
Final validation loss after ResNet-50 training as a function of input image representation and size. UTIraw: ultrasound as raw scanlines; UTIraw-padding: ultrasound as raw scanlines, reshaped to square; UTIwedge: ultrasound as a wedge shape.

Figure 5 presents the validation loss results that we obtained after training the ResNet-50 network separately for the three data representations as a function of the input image size. When comparing (1) raw data (*UTIraw*), (2) raw data in square form (*UTIraw-padding*), and (3) wedge-shaped ultrasound data (*UTIwedge*), we can see similar tendencies in the validation error (which is NMSE measured on the validation data). All of the errors with the raw scanlines and the wedge-formatted images are in the range of 0.44–0.55. The best results (lowest errors) were achieved with the (1) raw scanline representation. This is followed by the (2) raw data in square form, while the (3) wedge-shaped ultrasound data have the weakest results—but the values do not seem to be significantly different.

Therefore, we can conclude that the wedge representation of ultrasound tongue images (when extrapolated directly from the original raw scanlines) can result in roughly the same errors during articulatory-to-acoustic mapping.

### 3.3. Relation of Input Image Size and NMSE

We can investigate the three subfigures in Figure 5 as a function of image size. The tendencies are the same for all three data representations: the original image sizes (either 64×842 or 512×512 pixels) achieved a validation NMSE of around 0.48–0.49. When the image size is decreased (64×421 or 256×256 pixels), the validation error of the network will be lower. The optimal image size is around 64×64, resulting in a validation NMSE of around 0.44–0.45. Here, we can find some differences with the three data representations: (1) in the case of the raw scanline input (top subfigure), the image size causing the lowest error is 64×53 pixels; (2) if the scanlines are in square representation, then the lowest error is achieved with 256×256 pixels, but 64×64 results in almost the same values; (3) in the case of the wedge input, then, again, 64×64 pixels is the optimal size. If we further decrease the image size (64×26/64×13/32×32/etc.), then the error gets higher, until we reach the weakest results: NMSE is 0.45 with 64×13, and 0.52/0.56 with 8×8 pixels input images.

Based on the above comparison, we can conclude that the optimal image sizes are 64×53 and 64×64 when taking into account the validation error.

### 3.4. Training Time

**Figure 6:**
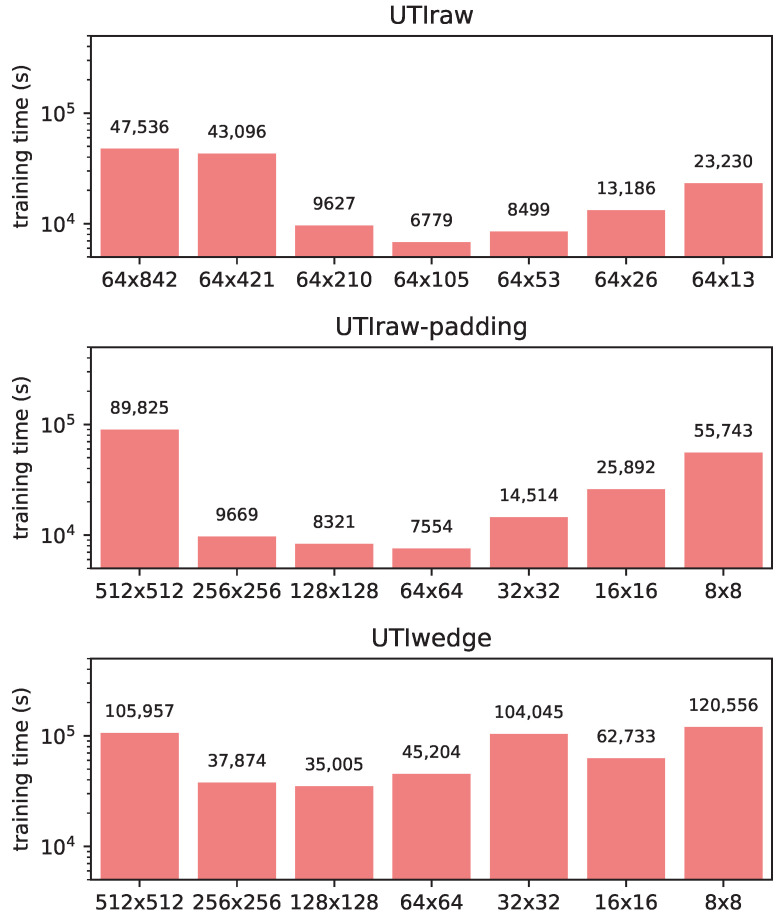
ResNet-50 wall-clock training time (in seconds) as a function of image size.

Figure 6 shows the (wall clock) DNN training times expressed in seconds. For all three input representations, this was measured on an Intel i7-2600 3.4 GHz PC with 16 GB RAM and an NVidia Titan X video card. Note that the largest images (512×512, 64×842, and 64×421) were trained with a batch size of 2 in order to fit into GPU memory; whereas, for the other image sizes, a batch size of 64 was used. The other parameters of DNN training were the same for all networks.

We can observe the tendency that networks with a middle-sized input image are faster to train. In particular, the original images (512×512 and 64×842) are highly disadvantageous when trained with ResNet-50 because of memory limitations (i.e., a smaller batch size). Based on the training time, the optimal image size is around 64×64 and 64×105 pixels (except for *UTIwedge*, where the training with the 128×128 input image size was the fastest). With *UTIwedge* representation, with all image sizes, the training time was significantly larger than with *UTIraw* or *UTIraw-padding*.

## 4. Discussion

For articulatory-to-acoustic mapping, ultrasound tongue imaging is often applied as an input, as shown in Section 1. Mostly, (micro)convex transducers are used, which provide a wedge-shape visual image. However, this is optimized for the visual inspection of the human eye (which is perfect for linguistic or medical studies), and the signal is often post-processed by the equipment (which might be a problem for engineering studies). Examples for such early systems are: Acoustic Imaging Performa 30 Hz ultrasound machine [4], Aloka SSD-1000 machine [6], Terason T3000 ultrasound [5,7,9], and the CONTEC CMS600P2 system [18].

With newer ultrasound equipment, it is now possible to gain full access to the raw scanline data (i.e., ultrasound echo return). A good example for this is the “Micro” system (developed by Telemed Ltd., Vilnius, Lithuania, and distributed by Articulate Instruments Ltd., Musselburgh, UK),which is available since 2016, and was also used for our recordings in the MTA-ELTE Lendület Lingual Articulation Research Group [11,17,50]. In addition, it was used for large-scale databases, such as UltraSuite [51] and UltraSuite-TaL [52]. The advantage of the “Micro” ultrasound equipment in this context is that we can use the data without any internal post-processing of the device, and the feature extraction can be left up to the machine learning algorithms. For other scenarios, e.g., automatic tongue contour tracking from ultrasound images, preprocessing the features has been shown to be useful [53], but, for contour tracking in the above study, deep learning approaches have not been used, which could help the feature learning.

The raw scanline data used in this study refer to the digitized, envelope-detected beam vectors of the “Micro” ultrasound system. When the ultrasound is recorded internally in the device, the envelopes of raw beamformed RF signals are generated from the delay and sum of channel signals. After further demodulation, low-pass filtering, and amplitude operation, the scanline data can be obtained, and the final B-mode images can also be generated by image processing and coordinate transformation. Therefore, the significant information differences should exist between the raw beamformed RF signals and raw scanline data or final B-mode images, rather than raw scanline data and final B-mode images. However, there is no control of beamforming in “Micro” and we cannot have access to the above RF signal (p.c., Articulate Instruments Ltd.). With other ultrasound equipment (e.g., “Art” system of Articulate Instruments Ltd.), one can record and process the RF output, but, in this case, the hardware synchronization with the speech signal has to be solved.

Although a large number of studies have already applied ultrasound tongue imaging for articulatory-to-acoustic mapping, the optimal data representations and input image sizes have not been deeply investigated before. In the current study, we compared the raw scanline representation (digitized, envelope-detected beam vectors) with the wedge-shaped processed UTI as the input for the residual network applied for AAM, and showed that all input representations can result in a similar validation error while training DNNs. We expect that, with a higher resolution ultrasound (e.g., higher fps, larger spatial resolution, or 3D/4D ultrasound [54]), the synthesized speech would be more natural, i.e., result in a lower MSE during DNN training.

However, a comparison across sessions and speakers (or designing speaker-independent AAM systems) using the raw scanline data is a challenge. Because of the convex transducer, the raw data do not correspond to the original mid-sagittal slice, and non-linear interpolation is necessary to transform into real-world orientation. Therefore, for comparisons across sessions and speakers, the wedge-shape ultrasound images might be more useful than the raw scanline data. By using tracing methods on wedge-shaped ultrasound images, it is also possible to obtain a raw-like data representation [55], but this conversion cannot revert the postprocessing methods of the equipment, and the back-and-forth conversion obviously leads to some data loss.

In spite of the significant achievements of the last decade, potential SSI applications seem to still be far away from a practically working scenario. Part of the reason is the lack of fully developed cross-session and cross-speaker methodologies. With some articulatory tracking devices, there have already been such experiments, e.g., signal normalization and model adaptation for sEMG [56,57], domain-adversarial DNN training [32], inter-speaker analysis for EOS [58], region of interest detection and cropping for lip video [43], and articulation adaptation using Procrustes matching with EMA [27]. Ultrasound-based SSI systems, however, might be less robust, as slight changes in probe positioning causes shifts and rotations in the resulting image [59,60]. Therefore, the results of the current study can help future cross-session and cross-speaker experiments.

## 5. Conclusions

In this study, we compared the raw scanline input with the wedge-shaped ultrasound tongue image representation. In addition, we investigated the optimal input image size of a residual network applied for articulatory-to-acoustic mapping. We found that there is no significant difference between using the raw data (either in original form or transposed to a square) and the wedge shape that is directly extrapolated from the raw data. We also found that the optimal pixel size is 64 × 64 when taking into account the validation loss and network training time. Therefore, it is not necessary to use the full original 64 × 842 pixels raw scanline, but a smaller image is enough, which allows for the building of smaller networks using less training data. In addition, the smaller image size enables the use of multiple consecutive input images [11] or a recurrent neural network [15], as already applied in our earlier work.

The advantage of fan/wedge-shaped data is that they correspond to the physical/spatial orientation of the speaking organs of the subject; therefore, comparisons across sessions and speakers are relatively easy. In the future, we plan to apply the raw-to-wedge conversion methods for experimenting with speaker-independent articulatory-to-acoustic systems in order to develop practically working silent speech interface applications.

The Keras implementations are accessible at https://github.com/BME-SmartLab/UTI-optimization, last accessed on 30 October 2022.

## Data Availability

Trained models can be downloaded at https://github.com/BME-SmartLab/UTI-optimization, last accessed on 30 October 2022.

## Abbreviations

The following abbreviations are used in this manuscript:

AAI	Acoustic-to-Articulatory Inversion
AAM	Articulatory-to-Acoustic Mapping
CNN	Convolutional Neural Network
DNN	Deep Neural Network
EMA	Electromagnetic Articulography
EOS	Electro-Optical Stomatography
LSTM	Long-Short Term Memory
MGC-LSP	Mel-Generalized Cepstrum–Line Spectral Pair
MGLSA	Mel-Generalized Log Spectral Approximation
PMA	Permanent Magnetic Articulography
sEMG	surface Electromyography
SSI	Silent Speech Interface
SSR	Silent Speech Recognition
TTS	Text-To-Speech
UTI	Ultrasound Tongue Imaging
